# Carbon Nanostructure of Kraft Lignin Thermally Treated at 500 to 1000 °C

**DOI:** 10.3390/ma10080975

**Published:** 2017-08-21

**Authors:** Xuefeng Zhang, Qiangu Yan, Weiqi Leng, Jinghao Li, Jilei Zhang, Zhiyong Cai, El Barbary Hassan

**Affiliations:** 1 Department of Sustainable Bioproducts, Mississippi State University 1, Starkville, MS 39759, USA; njfuxf@gmail.com (X.Z.); e.hassan@msstate.edu (E.B.H.); 2 USA Department of Agriculture, Forest Service, Forest Products Laboratory, Madison, WI 53726, USA; Weiqileng@gmail.com (W.L.); csuftljh@gmail.com (J.L.)

**Keywords:** kraft lignin, thermal treatment, nanocrystallites, structural parameters, amorphous carbon

## Abstract

Kraft lignin (KL) was thermally treated at 500 to 1000 °C in an inert atmosphere. Carbon nanostructure parameters of thermally treated KL in terms of amorphous carbon fraction, aromaticity, and carbon nanocrystallites lateral size (*L*_a_), thickness (*L*_c_), and interlayer space (*d*_002_) were analyzed quantitatively using X-ray diffraction, Raman spectroscopy, and high-resolution transmission electron microscopy. Experimental results indicated that increasing temperature reduced amorphous carbon but increased aromaticity in thermally treated KL materials. The *L*_c_ value of thermally treated KL materials averaged 0.85 nm and did not change with temperature. The *d*_002_ value decreased from 3.56 Å at 500 °C to 3.49 Å at 1000 °C. The *L*_a_ value increased from 0.7 to 1.4 nm as temperature increased from 500 to 1000 °C. A nanostructure model was proposed to describe thermally treated KL under 1000 °C. The thermal stability of heat treated KL increased with temperature rising from 500 to 800 °C.

## 1. Introduction

Lignin is one of the most abundant natural polymers and accounts for 10–35 dry wt % of total 200 × 10^9^ tons/year of global lignocellulosic biomass production [1,2]. The main source of lignin comes from wood pulping and cellulosic ethanol industries as byproducts [3,4]. The global annual production of lignin from the wood pulping process is around 70 million tons [5]. However, the majority of lignin, especially kraft lignin (KL, 80% of the world’s chemical pulping lignin production) is burned onsite for energy and pulping chemicals recovery, and less than 2% of lignin is isolated from pulping liqueurs and used for value-added products [6]. As a highly functional aromatic polymer, lignin was considered as a good candidate for the substitution of polyol and phenol for synthesizing polyurethanes [7] and phenolic resins [8], however, its large-scale application is limited because of its heterogeneous structure and low reaction activity.

Recently, thermal treatment of lignin is widely investigated for producing fuels and chemicals [3,9], but the high process cost is the major challenge for the commercialization of these technologies. Thermal treatment of lignin also has been researched for producing solid carbon products such as activated carbon [10] and carbon fiber [11] because of a high carbon content of lignin (>60 wt %). The physicochemical properties of activated carbons and carbon fibers are dominated by their nanostructures. Thermal treatment of lignin yields non-graphitizing carbons consisting of many nanocrystallites (nano-sized graphene layers) cross-linked with amorphous carbon [12], and these non-graphitizing carbon cannot be converted to graphite even at 3000 °C. The structural properties of non-graphitizing carbons such as lateral size (*L*_a_) and thickness (*L*_c_) of nanocrystallites, and amorphous carbon fraction (*X*_a_) are critical to their chemical and physical properties, and these properties are affected by process temperature [13]. Limited literature is found related to quantitative analyses of these structural properties as a function of thermal treatment conditions such as temperature under 1000 °C for lignin, especially, KL.

The common methods used to analyze carbon structures are X-ray diffraction (XRD), Raman spectra, Fourier transform infrared spectroscopy (FTIR), and high-resolution transmission electron microscopy (HRTEM) image processing. Among these mentioned characterization techniques, XRD and Raman provide quantitative information about carbon structural lateral sizes like *L*_a_ and *L*_c_. Rodríguez-Mirasol et al. used XRD methods to analyze *L*_c_ of carbons in KL treated at 1100 to 2400 °C [14]. Later, their group used Raman characterized electrospun Alcell lignin-based carbon fiber produced at temperatures between 600 to 1000 °C, and the results suggested that the degree of structural organization on those lignin-based carbon fibers was superior to the reported pitch-based carbon fibers prepared at same temperature levels [15,16]. Lu et al. characterized *X*_a_, *L*_a_, *L*_c_, and *d*_002_ of various coals using the XRD method, and standardized their quantitative analysis procedures for disordered carbons [17]. Zickler et al. discussed the differences between *L*_a_ values determined from XRD and Raman methods for thermally treated wood [18]. FTIR is used studied chemical structural changes of alkali lignin heated at 150 to 550 °C [19] and chemical pathway of the aromatic structure of lignin thermal treated at 300 to 1400 °C [20]. The HRTEM image processing method is widely used for quantitative analyses of the nanocrystalline structure of various carbon materials such as coals [21,22], carbon black [23], soot [24], and thermally treated polymers [25].

In this study, KL was thermally treated at six temperature levels (500, 600, 700, 800, 900 and 1000 °C). Temperature effects on carbon nanostructures of these thermally treated KL in terms of lateral size (*L*_a_) and thickness (*L*_c_) of nanocrystallites and amorphous carbon fraction (*X*_a_) were characterized quantitatively using FTIR, XRD, Raman, and HRTEM image processing methods. The potential application of carbon nanomaterials produced could be the raw material for making battery anodes [26].

## 2. Materials and Methods

### 2.1. Thermal Treatment Process

Deionized-water purified softwood KL was supplied by Domtar Corp. (Plymouth, NC, USA), its commercial name is Bio-Choice lignin. The specification provided by Domtar showed that KL contained 97.1% lignin and 1.7% sugar, and had a pH value of 6.2. The KL ash content measured in our lab was 0.53%, following ASTM D1102 [27]. The KL sample was sent to Soil Testing Laboratory of Mississippi State University for element analysis, where carbon, hydrogen, and oxygen content were measured by a PE 2400 CHN Elemental Analyzer (PerkinElmer, Houston, TX, USA), and inorganic components was measure by a 4300 Optima ICP Spectrophotometer (PerkinElmer, USA). The oxygen content was evaluated by the difference, and the element analysis results of KL is shown in Table 1.

The effects of six temperature levels (500, 600, 700, 800, 900, and 1000 °C) on the nanostructure of thermally treated KL samples were investigated. For each target temperature run, four grams of KL were loaded respectively on two porcelain boats (each holds 2 g) placed in the heating zone of a split-hinge 50 mm-quartz tube electric furnace (Lindberg/Blue M 1200. Thermo Scientific™, Pittsburgh, PA, USA) equipped with a temperature controller (Lindberg/Blue UTC 150). After purging argon gas (99.99%) for 15 min to exclude air from the system, the target temperature was raised to at a ramping rate of 20 °C/min under atmospheric pressure with an argon gas (99.99%) at a flow rate of 1.8 L/min. After holding each evaluated temperature for 1 h, the furnace was turned off and allowed to cool to ambient temperature under an argon atmosphere. Each thermally treated KL sample was removed out from the heating system, weighed, labeled as KL-X, where X represents the evaluated temperature level, for instance, KL-1000 represents the KL sample treated at 1000 °C. In order to study the KL thermal degradation process, KL was also heated at 200, 300, 400 °C for 2 h under the same thermal treatment condition. All sample solid carbon materials yields (*Y*, %) were calculated using the Equation (1).

(1) $$Y=\frac{W_{1}-W_{\mathrm{ash}}}{W_{0}-W_{\mathrm{ash}}}\times 100\%$$

where *W*_1_ is the solid materials weight after thermal treatment (g), *W*_0_ is oven-dried kraft lignin weight (g), *W*_ash_ is ash weight (g).

In addition to the thermal treatment of KL samples using the described furnace system, the thermogravimetric analysis (TGA) was conducted to analysis KL thermal decomposition behavior. TGA experiment was performed on a thermo-gravimetric analyzer (TGA Q5000, TA Instruments, New Castle, DE, USA), 5 mg of KL was first pretreated in the argon (99.99%) flow at 25 °C for 5 min, then heated up to 1000 °C at a heating rate of 10 °C/min under argon flow (100 mL min^−1^).

### 2.2. Characterization

#### 2.2.1. FTIR

The FTIR spectra of KL-X samples were recorded with the PerkinElmer attenuated total reflection (ATR) spectrometer (PerkinElmer, USA) at a resolution of 2 cm^−1^ for 10 scans in the 450 to 4000 cm^−1^ range. Powdered KL-X samples were pressed against the ATR device diamond crystal. The background spectrum of air was subtracted from the sample spectrum. The spectra were baseline-corrected by applied “Data Tune-up” using Spectrum^®^ Quant software (PerkinElmer, USA). Five replicates were analyzed for each of six KL-X samples.

#### 2.2.2. XRD

XRD analyses of all KL-X samples, one sample per temperature level, were conducted on Ultima3 diffractometer (Rigaku, Japan. CuKα radiation with *λ* = 1.5406 Å). The diffraction intensities were measured in the 2*θ* range from 3 to 120° on a step-scan mode (0.1°/step), and the intensities were collected for 6 s at each step. The quantitative analysis of XRD patterns was conducted using the method developed by Lu et al. The original XRD data sets of KL-X samples were first converted into reduced intensity XRD patterns through polarization correction, intensity normalization, and reduction steps. Three (0 0 2), (1 0), and (1 1 0) graphite diffraction peaks appeared in the reduced intensity patterns were used to calculate five structural parameters of KL-X samples, including amorphous carbon fraction (*X*_a_), aromaticity (*f*_a_), interlayer spacing (*d*_002_), nano-crystallite lateral size (*L*_a_) and thickness (*L*_c_).

The *X*_a_ value was resolved by adjusting the (002) peak to the most symmetrical peak. The value of *f*_a_ describing the ratio of carbon atoms in edge aliphatic chains *vs* aromatic rings was calculated by the deconvolution of 2*θ* region of 15–37° to two pseudo-Voigt peaks around 20° (*γ* peak) and 26° ((0 0 2) peak) using the Equation (2) [17]:

(2) $$f_{a}=\frac{A_{002}}{A_{002}+A_{\gamma }}$$

where *A*_002_ and *A_γ_* are the areas underneath of (0 0 2) and *γ* peaks, respectively.

The *d*_002_ value (Å) of carbon nanocrystallites was calculated from the (0 0 2) peak by using the Bragg equation [28]:

(3) $$d_{002}=\frac{\lambda }{2\sin\theta }$$

where *λ* is the wavelength (Å), *θ* is the Bragg angle of the (0 0 2) peak.

*L*_a_ and *L*_c_ values of carbon nanocrystallites were calculated from (0 0 2) and (1 1 0) peaks using the following Scherrer equation [29]:

(4) $$L_{a/c}=\frac{K\lambda }{B\cos\theta }$$

where *λ* is the wavelength (nm); *B* and *θ* correspond to the full width at half maximum (FWHM) and the Bragg angle of the corresponding peak, respectively; *K* is equal to 0.89 and 1.84 for (0 0 2) and (1 1) peaks, respectively. More detailed information regarding these structural parameters calculations can be found in the dissertation [30].

#### 2.2.3. Raman

Raman spectra of all KL-X samples, one sample per temperature level, were recorded using a LabRAM Arimas confocal Raman microscope (Horiba, Japan) under a green laser (wavelength *λ* = 532 nm). The spectra were deconvoluted into four peaks (corresponding to ~1170 cm^−1^, D1, D3, and G bands, respectively) by pseudo-Voigt function [15,18]. The band intensities, including both peak height intensity (*I*) and peak area intensity (*A*), were resolved from Lorentz peaks. The intensity ratios of both D3 to G peak and D (D1 plus D3) to G peak were calculated, i.e., *I*_D_/*I*_G_ represents the height ratio of D to G peak, and *A*_D_/*A*_G_ represents the integrated area ratio of D to G peak. The *L*_a_ value (nm) was calculated based on *I*_D_/*I*_G_ and *A*_D_/*A*_G_ using the following equations [18,29,31]:

(5) $$L_{a,I}=(C_{0}+\lambda C_{1}){\left(\frac{I_{D}}{I_{G}}\right)}^{-1}$$

(6) $$L_{a,A}=(C_{0}+\lambda C_{1}){\left(\frac{A_{D}}{A_{G}}\right)}^{-1}$$

where *C*_0_ and *C*_1_ are wavelength pre-factor and *C*_0_ = −12.6 nm and *C*_1_ = 0.333, *λ* is the wavelength (nm).

#### 2.2.4. HRTEM

The nanostructure of KL-X samples was analyzed on a JEOL 2100 HRTEM (JEOL, Peabody, MA, USA) using at least 400,000 magnification. The KL-X samples were hand-ground to fine powder in ethanol and then sprayed over a lacey copper grid. Bright-field HRTEM images were analyzed using the methods proposed by previous studies [20,21,22,27,32]. The ImageJ software (available at https://imagej.nih.gov/ij/) was used for image processing, and the detailed processing procedures can be found in the dissertation [30]. The digitized HRTEM image was first converted to a negative image after contrast enhancement, followed by the filtration procedure of using the Gaussian filter. The filtered image was converted to a skeletonized image to extract carbon lattice layers. The extracted carbon layers were subjected to morphological modification to repair aggregated layers, followed by converting the image to a black-and-white skeletonized image. The average length of carbon layers (*L*_a_) was calculated from software outputs. Carbon-based materials with their layer lengths less than 0.5 nm were considered as amorphous carbon and discarded from *L*_a_ calculation.

## 3. Results and Discussion

### 3.1. Thermal Treatment Yield

**Figure 1:**
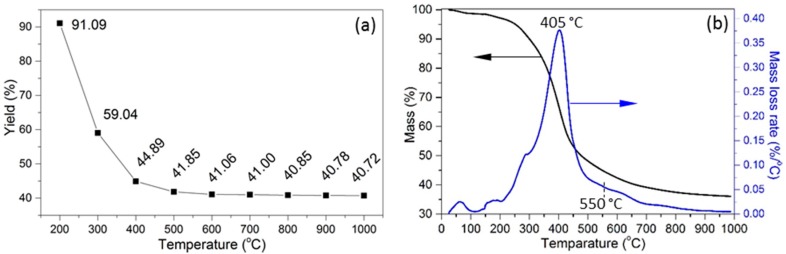
Thermal analysis of KL (**a**) KL-X yield at different temperatures; (**b**) TG and DTG curves.

The solid yield of KL-X samples decreased from 91.09 to 44.89% as the temperature increased from 200 to 400 °C (Figure 1a). The yield of KL-X samples kept around 41% as the temperature increased from 500 to 1000 °C. Three major weigh loss stages were observed in thermogravimetric (TG) and differential thermogravimetric (DTG) curves (Figure 1b). The first stage had 1.5% weight loss as the temperature increased from 30 to 150 °C because of the evaporation of free water. The major KL weight loss of 53.6% occurred at the second stage as the temperature increased from 150 to 550 °C because of its thermal decomposition, and its maximum weight loss rate of 0.38 wt %/°C was attained at 405 °C. The minor shoulder DTG peak at 320 °C can be attributed to decomposition of sugar impurity in KL. The weight loss of KL in the third stage between 550 and 1000 °C was 8.8%. The calculated yield based on the thermal treatment performed in the furnace was 41.85% at 500 °C, which was different from the yield result of 48.18% from the TGA test. The inconsistency was because TGA provided the real-time yield at 500 °C, while the furnace system provided the yield of KL sample treated at 500 °C for 2 h.

### 3.2. Characterization of Solid Carbon Structure

#### 3.2.1. FTIR

**Figure 2:**
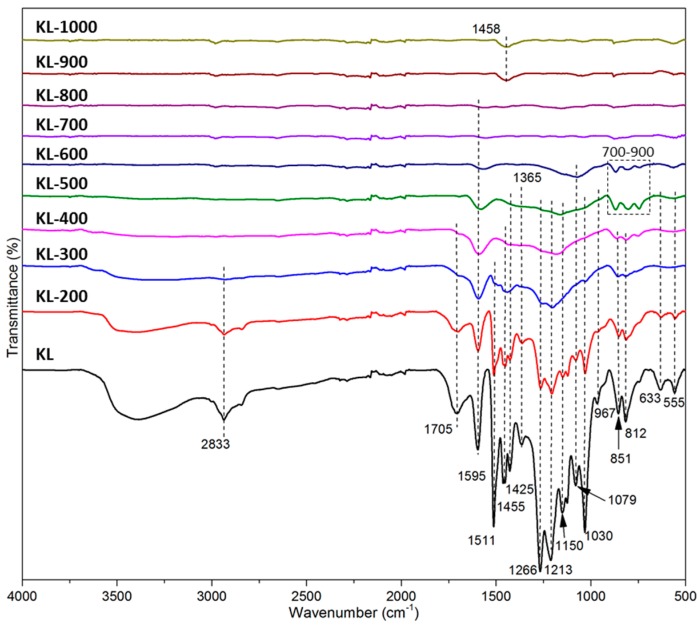
Fourier transform infrared (FTIR) spectra of KL-X samples.

FTIR spectra (Figure 2) show that the reduction of infrared (IR) signals of KL-X samples mainly occurred at the temperature range of 200 to 700 °C. The reduction of IR signals at the temperature range of 200 to 500 °C was significantly higher than that of 500 to 700 °C. This suggested the majority of lignin side chains were decomposed after heated at 500 °C for 2 h. Specifically, the reduction of 1705 cm^−1^ (corresponding to C=O) [33], 1079 and 1030 cm^−1^ (both corresponding to aliphatic C–O) bands [34] in the temperature range from 200 to 500 °C can be attributed to cracking and reforming of carboxyl (–COO–), carbonyl (–CO–) [35], and ether (R–O–R) groups. The reduction of IR bands at 3600~3100 cm^−1^ (corresponding to –OH) [36] and 2860~2939 cm^−1^ (corresponding to –CH_n_) [37] at the temperature below 400 °C resulted from lignin dehydration and decomposition of aliphatic chains [38]. Besides, the reduction of 633 and 555 cm^−1^ IR bands at the temperature below 300 °C was related to the decomposition of sugar (hemicellulose) impurities. All IR bands for aliphatic chains (1079 cm^−1^), methoxyl (1425 cm^−1^), and aromatic-bonded oxygen (1266, 1365 cm^−1^) groups [34,39] were considerably reduced as temperature increased to 500 °C. The decreases of aromatic C=C stretch and C–H deformation at 1595 cm^−1^ and 1150 cm^−1^, respectively [40], indicated the increase of aromatic condensation degree [41]. However, the high intensity between 700~900 cm^−1^ appeared the spectrum of KL-500 suggested the formation of volatile tars on the solid carbon surface at 500 °C [19]. The intensity of IR band at 700~900 cm^−1^ decreased with the rise of temperature from 500 to 700 °C indicated the secondary pyrolysis of tars [35]. As temperature further increased to 700 and 800 °C, IR bands became nearly invisible in KL-X samples, except for the weak aromatic C=C stretch band near 1595 cm^−1^, suggested nearly complete decomposition of side chains below 800 °C. Meanwhile, the dehydrogenation and rearrangement of aromatic rings probably started at this temperature range, led to the increase of aromatic condensation degree, i.e., the growth of nano-graphene layers sizes [20]. New IR bands near 1458 cm^−1^ (C=C–O deformation) appeared in KL-900 and KL-1000 spectra can be attributed to the dehydrogenation and cracking of aromatic rings [20], which possibly resulted in the rearrangement of nano-graphene layers.

#### 3.2.2. XRD

**Figure 3:**
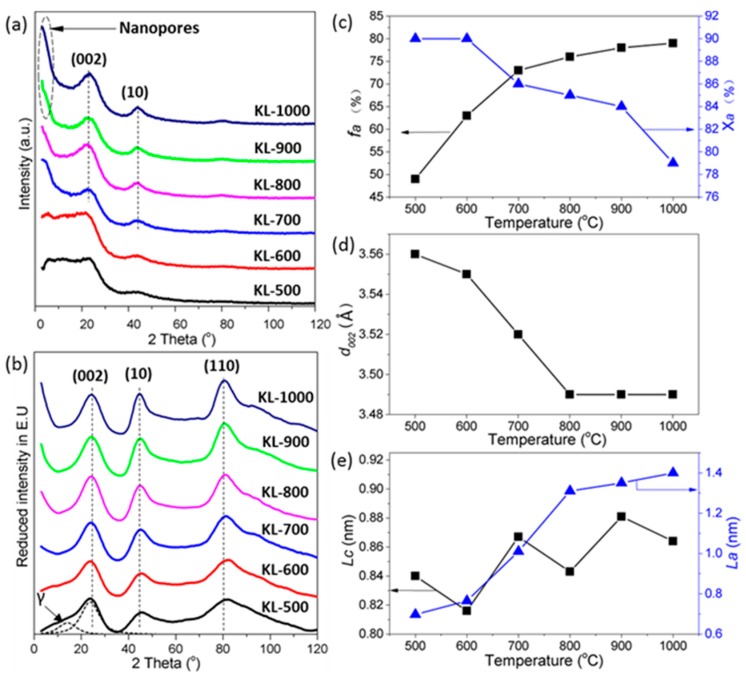
X-ray diffraction (XRD) analysis of KL-X samples: (**a**) wide angle (2*θ* = 3~120°) XRD patterns; and (**b**) reduced intensity XRD patterns; (**c**–**e**) the influence of heating temperature on the structural parameters of KL-X samples; (**c**) aromaticity *f*_a_ and amorphous carbon fraction *X*_a_; (**d**) interlayer spacing *d*_002_; and (**e**) nanocrystallite lateral size *L*_a_ and thickness *L*_c_.

There was no diffraction peak detected in XRD patterns (Figure 3a) of KL-500 and KL-600 samples, indicating that these thermally treated KL samples had disorder structures. Diffraction peaks appearing around 23° and 42° in KL-X samples treated above 700 °C can be attributed to (0 0 2) and (1 0) reflections of carbon nanocrystallites [42], respectively. Increasing the intensity at diffraction angle less than 10° within the temperature range from 700 to 1000 °C suggested nanopores developed in KL-X samples [43].

The values of structural parameters (*X*_a_, *f*_a_, *d*_002_, *L*_a_, and *L*_c_) of carbon nanocrystallites were calculated using reduced intensity XRD patterns (Figure 3b), summarized in Table 2, and were also plotted as a function of temperature in Figure 3c–e, respectively. In general, *f*_a_ values increased as the temperature increased from 500 to 1000 °C, but the growth ratios were different in different temperature ranges. The growth ratio in the lower temperature range (500 to 700 °C) was 0.12%/°C, which was faster than 0.02%/°C in the higher temperature range (700 to 1000 °C). There were three decrease ratios of 0.04, 0.01, and 0.05%/°C observed in three temperature ranges from 600 to 700 °C, 700 to 900 °C, and 900 to 1000 °C for *X*_a_ values, respectively. All these results indicated that there was an increasing trend of KL-X structural ordering as the temperature increased from 500 to 1000 °C, but the magnitude of growth ratios was affected by temperature. The *d*_002_ value decreased with the rise of temperature from 500 to 800 °C (Figure 3d) and no further decrease was observed as the temperature further increased from 800 to 1000 °C. The decreasing ratio in the lower temperature range from 500 to 600 °C was 1 × 10^−4^ Å/°C, which was lower than 3 × 10^−4^ Å/°C in the higher temperature range from 600 to 800 °C. All KL-X samples having *d*_002_ values larger than 3.35 Å (graphite lattice distance) indicated that the majority of nano-graphene layers in KL-X samples stacked in turbostratic structure [44]. The *L*_a_ value of carbon nanocrystallites in KL-X samples grew at a faster rate of 2.04 × 10^−2^ nm/°C as the temperature increased from 500 to 800 °C compared to the rate of 4.4 × 10^−3^ nm/°C as the temperature further increased from 800 to 1000 °C (Figure 3e). The increase of *L*_a_ was mainly because of the decomposition of lignin side chains [45] and the aromatization of free radical contained aromatic groups, respectively. This was evidenced by FTIR results (Figure 2) indicating complete thermal creaking of lignin side chains occurred at temperature below 800 °C. There was an increasing trend for *L*_c_ as the temperature increased from 500 to 1000 °C, but the increase had some up and down fluctuation. Aso et al. also observed that the growth of *L*_a_ was faster than *L*_c_ for the thermally treated polyfurfuryl alcohol-derived carbons.

#### 3.2.3. Raman

**Figure 4:**
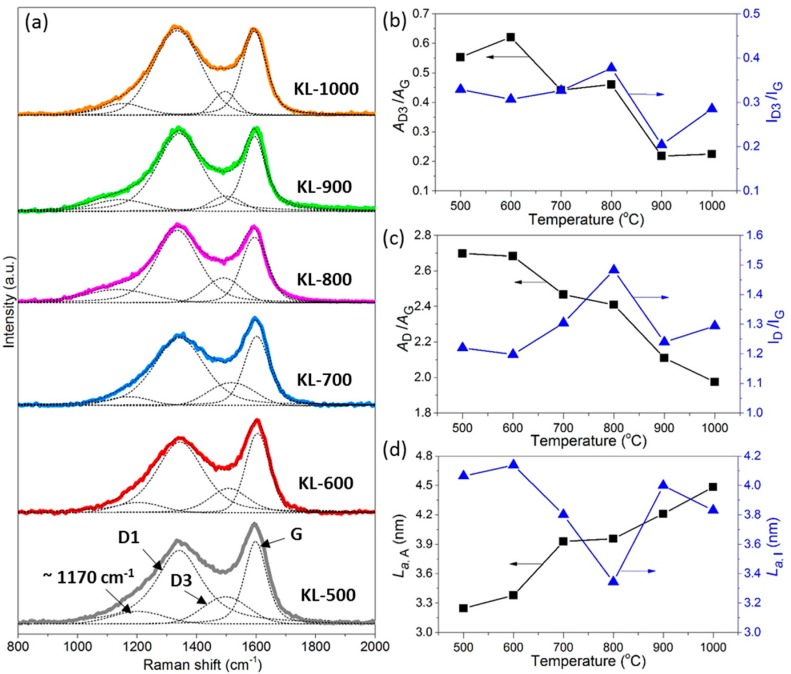
Raman analysis of KL-X samples: (**a**) Raman spectra for KL-X samples; (**b**–**d**) the influence of heating temperature on the structural parameters of KL-X samples; (**b**) the area and height ratio *I*_D3_/*I*_G_ of the D3 and G bands; (**c**) the area and height ratio *I*_D_/*I*_G_ of the D and G bands; and (**d**) the nanocrystallite lateral size *L*_a_ calculated from area and height intensity of D and G bands.

Raman spectra (Figure 4a) of KL-X samples were deconvoluted into four pseudo-Voigt shaped peaks centered at ~1170, ~1345 (D1 band), 1500 (D3 band), and 1600 cm^−1^ (G band), respectively. The D1, G, and D3 bands indicated *sp*^2^ bonded turbostratic carbon nanocrystallites [46], graphitic carbon nanocrystallites, and amorphous carbon [47], respectively. The band at ~1170 cm^−1^ was related to ions impurities, i.e., lignin ash, and interstitial defects [15].

In general, the calculated values of *A*_D3_/*A*_G_ and *A*_D_/*A*_G_ had a decreasing trend as temperature increased from 500 to 1000 °C, while there was no predictable pattern in how temperature affected *I*_D3_/*I*_G_ and *I*_D_/*I*_G_ values (Figure 4b,c). The average decreasing rates of *A*_D3_/*A*_G_ and *A*_D_/*A*_G_ were 6.6 × 10^−4^/°C and 1.46 × 10^−3^/°C from 500 to 1000 °C. These decreases confirmed the reduction of the amorphous carbon fraction [47] and the increased structural ordering of the KL-X samples [48]. The calculated *L*_a-A_ values increased with the rise in temperature from 500 to 1000 °C, however, there was no increasing trend observed for *L*_a-I_ values with the rise of temperature (Figure 4d). The increasing trend of *L*_a-A_ values matching to XRD results suggested that *A*_D_/*A*_G_ is more suitable for calculation of *L*_a_ than *I*_D_/*I*_G_ when used for KL-X samples’ structure parameter calculation. This observation was different from the report from Cançado et al. indicating that *I*_D_/*I*_G_ is more suitable for *L*_a_ calculation than *A*_D_/*A*_G_ for disorder carbon materials [31].

#### 3.2.4. HRTEM

**Figure 5:**
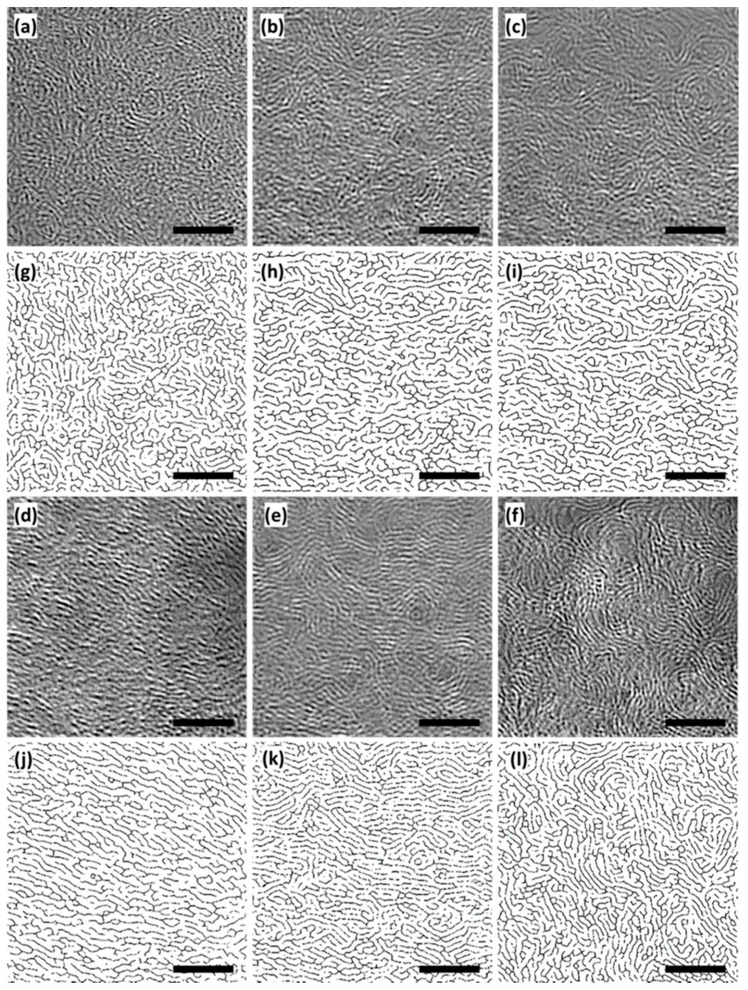
High-resolution transmission electron microscopy (HRTEM) analysis of KL-X samples: the negative HRTEM images of (**a**) KL-500; (**b**) KL-600; (**c**) KL-700; (**d**) KL-800; (**e**) KL-900; (**f**) KL-1000 showing the morphologies of nano-graphene layers; and (**g**–**l**) are the corresponding skeletonized HRTEM images showing the arrangements and lateral size *L*_a_ of nano-graphene layers. The size bars represent 5 nm.

The negative bright-field HRTEM images of KL-X samples (Figure 5a–f) illustrated that bright fringes were nano-graphene layers, and dark spots were structure defects and nanopores. The length of fringes represented the lateral size of nano-graphene (*L*_a_). The HRTEM images also show that the arrangement of fringe was nondirective, and the longer fringes are more likely intertwined together. Figure 5g–l shows the corresponding skeletonized HRTEM images of KL-X samples. Table 1 summarizes the average fringe length values of KL-X samples measured and calculated using the ImageJ software. The average fringe length was increased as temperature increased from 500 to 900 °C, while it decreased at 1000 °C. This phenomenon was probably because of the growth of nano-graphene fringes was nondirective, and the fringes were intertwined together. Two across fringes in HRTEM image was considered to be four respective fringes by ImageJ software, and which resulted in the calculated length shorter than its actual length (especially for the large fringes).

All characterization techniques show that *L*_a_ values increased with the temperature. However, the *L*_a_ values calculated from each of three techniques differ. The difference in the *L*_a_ values is partly because of the difference in theories and principles on which these techniques are based [49]. The HRTEM method determines the graphene layer size, while the XRD and Raman values determine the carbon nanocrystallite sizes. Logically, the *L*_a_ value calculated from the Raman technique should be close to the XRD technique and smaller than the HRTEM technique. However, the difference in *L*_a_ values calculated from XRD and Raman techniques is probably because of the difference in the principles of XRD and Raman spectra. The *L*_a_ value calculated by the HRTEM technique was smaller than the XRD technique for KL-700, KL-800, KL-900, and KL-1000, this was because high temperature treated samples (700 to 1000 °C) contains more intertwined graphene layers, and their length was underestimated during the image processing process.

#### 3.2.5. Thermal Stability

**Figure 6:**
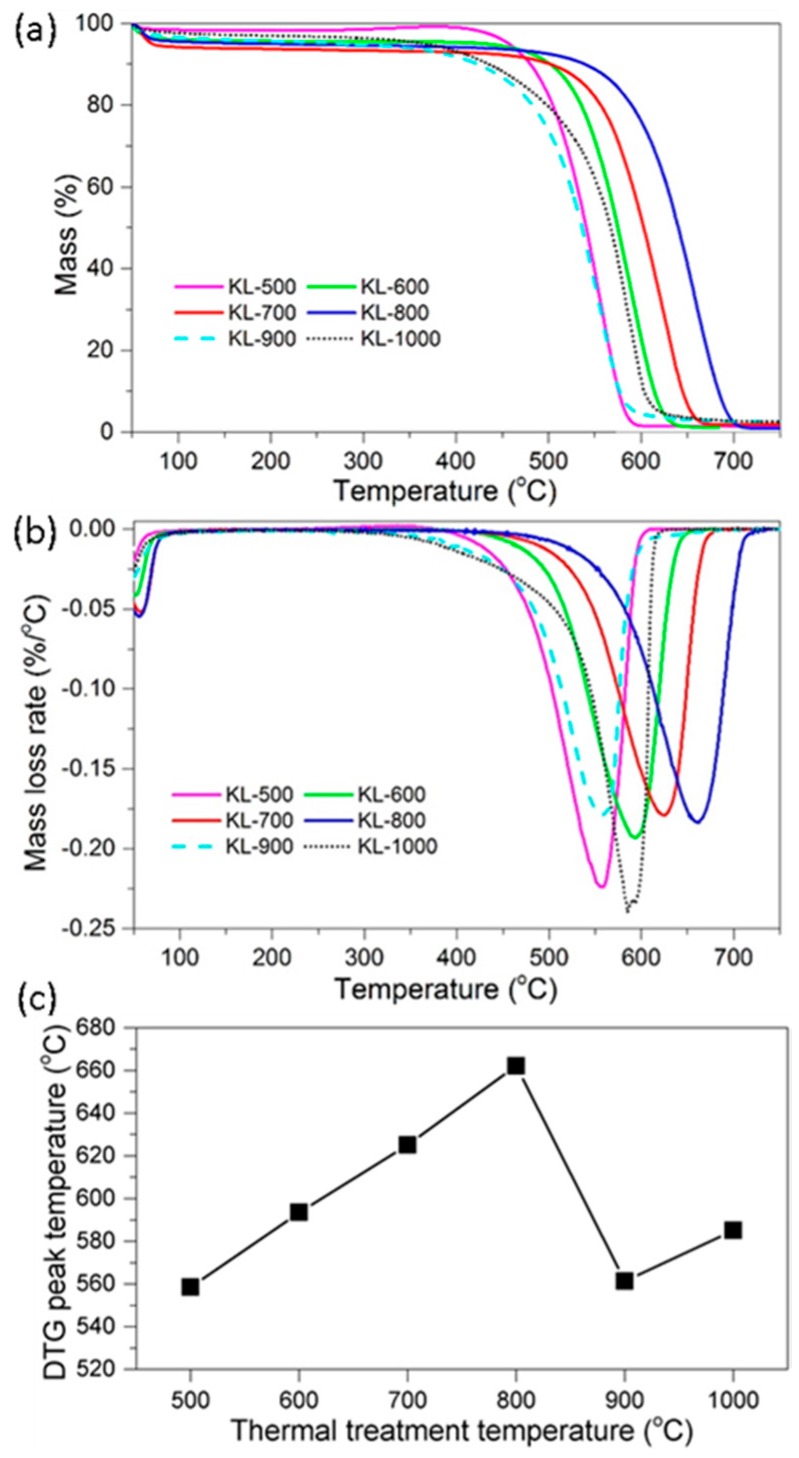
Thermal stability analysis of KL-X samples: (**a**) mass loss TG and (**b**) mass loss rate DTG curves of KL-X samples oxidized in air atmosphere; and (**c**) and influence of KL-X samples production temperature on its DTG peak temperature.

TG and DTG curves of KL-X samples at a heating rate of 10 °C/min under an air flow are shown in Figure 6a,b, respectively. The peak of a mass loss rate shows in DTG curve (DTG peak) was located in a broad temperature region (between 533 and 663 °C).

The DTG peak temperature of KL-X samples was plotted versus the thermal treatment temperature of KL samples (Figure 6c). As the thermal treatment temperature of lignin increased from 500 to 800 °C, the DTG peak temperature increased from 558.4 to 662.1 °C, suggested the thermal stability of KL-X samples increased with the rise of temperature from 500 to 800 °C. This was because KL-X samples treated at a higher temperature contained less amorphous carbon. KL-900 and KL-1000 samples had lower DTG peak temperatures than KL-800, KL-700, and KL-600 samples, suggesting that KL samples treated at a higher temperature were less stable for thermal oxidation if compared to a lower temperature. This phenomenon can be attributed to KL-900 and KL-1000 containing more nanopores in their structures, as described in our XRD results. Xie et al. reported that carbons in organosolv lignin treated at 1000 °C contained a significantly higher amount of nanopores (2~75 nm in diameter) than those treated at 700 °C [41]. Aso et al. reported that carbons derived from heat treating polyvinyl chloride (PVC) and polyfurfuryl alcohol (PFA) at 1000 °C had a lower oxidative decomposition peak temperature than those at 900 °C [25]. Ruiz-Rosas et al. investigated the oxidation resistance of lignin-based carbon fiber with and without platinum, they found carbon fiber without platinum produced at the temperature of 1000 °C had lower oxidation resistance than that of 900 °C, and this was because of the development of nanopores and surface area at 1000 °C [15]. Besides, the presence of platinum decreases the oxidation resistance of lignin-based carbon fiber because platinum widened the nanopore structure of carbon fiber [15]. KL is an oxygen-rich aromatic compound compared to PVC and PVA materials. Therefore, more nanopores can be developed during thermal treatment. In addition, the presence of inorganic ash in KL can also lead to the development nanopores at high temperatures (900 to 1000 °C).

### 3.3. Carbon Nanostructure Model of KL-X

**Figure 7:**
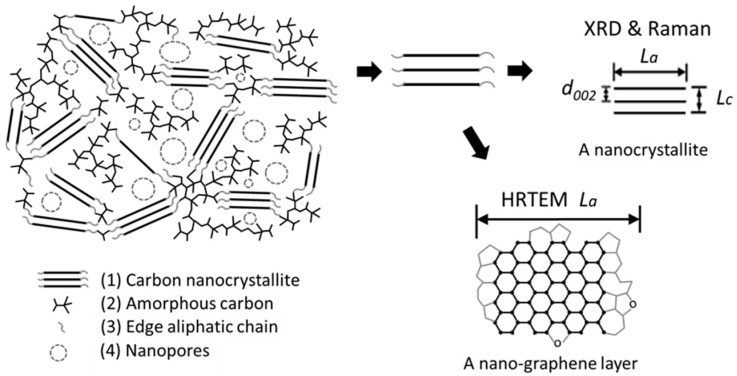
Schematic image of the carbon nanostructure model of KL-X samples. The KL-X samples consisted of carbon nanocrystallites, amorphous carbon, and nanopores. The nanocrystallites consisted of 2–4 layers of turbostratic stacked nano-graphene. The lateral size, thickness, and interlayer space of nano-graphene are *L*_a_, *L*_c_, and *d*_002_, respectively.

A carbon nanostructure model (Figure 7) was proposed to describe the structural components in KL-X samples based on above characterization results. The model consisted of carbon nanocrystallites, amorphous carbon, and nanopores. The carbon nanocrystallites is a series of turbostratic stacked nano-graphene layers with many aliphatic side chains attached on their edges [12,50]. The amorphous carbon was a mixture of randomly hybridized non-aromatic carbon atoms trapped in carbon nanocrystallites. The nanopores spread within the carbon nanocrystallites and amorphous carbon mixture.

## 4. Conclusions

The carbon nanostructure of KL thermally treated at 500 to 1000 °C was quantitatively analyzed. A nanostructure model was proposed for thermally treated KL. The structural ordering of thermally treated KL increased with the rise of heating temperature from 500 to 1000 °C. The increased structural ordering of thermally treated KL samples led to the reduction of their oxidation activity in the temperature range from 500 to 800 °C, while the development of nanopores in thermally treated KL samples resulted in the rise of their oxidation activity in the temperature range from 800 to 1000 °C.

## Figures and Tables

**Table 1:** Element analysis results of Kraft lignin (KL).

C	H	O	N	P	K	Ca	Mg	S	Fe	Mn	Zn	Cu	B
(%)									(ppm)				
63.00	5.78	30.79	0.17	0.02	0.07	0.04	0.01	0.12	19	27	16	1	19

**Table 2:** Calculated structural parameters of KL-X samples from XRD, Raman, and HRTEM analysis.

Sample	XRD					Raman						HRTEM
	*X*_a_ (%)	*f*_a_ (%)	*d*_002_ (Å)	*L*_c_ (nm)	*L*_a_ (nm)	*A*_D3_/*A*_G_	*I*_D3_/*I*_G_	*A*_D_/*A*_G_	*I*_D_/*I*_G_	*L*_a,A_	*L*_a,I_	*L*_a_ (nm)
										(nm)		
KL-500	90	49	3.56	0.84	0.70	0.55	0.33	2.70	1.22	3.24	4.06	0.71
KL-600	90	63	3.55	0.82	0.77	0.62	0.31	2.68	1.20	3.38	4.14	0.79
KL-700	86	73	3.52	0.87	1.01	0.44	0.33	2.47	1.30	3.93	3.80	0.86
KL-800	85	76	3.49	0.84	1.31	0.46	0.38	2.41	1.48	3.96	3.34	0.94
KL-900	84	78	3.49	0.88	1.35	0.22	0.20	2.11	1.24	4.21	4.00	1.06
KL-1000	79	79	3.49	0.86	1.40	0.22	0.29	1.97	2.29	4.48	3.83	0.99
